# Supplementary material for: Rapid memory encoding in a recurrent network model with behavioral time scale synaptic plasticity
Source: PLoS Comput Biol. 2023 Aug 25;19(8):e1011139. doi: 10.1371/journal.pcbi.1011139 (PMC10484462; doi:10.1371/journal.pcbi.1011139)
Supplement: S1 Appendix — (PDF) [file pcbi.1011139.s001.pdf]

Detailed calculation of memory traces and quenched variability in the BTSP rule

We start with the 1D map for BTSP in a recurrent network with sparse coding, Eq.29 from the Methods. We rewrite the equation as

$$\begin{aligned} w_{ij}^n &= Pf_P(\Delta\theta_{ij}^n)S_i^nS_j^n + w_{ij}^{n-1}\big(1-(Pf_p(\Delta\theta_{ij}^n)+Df_D(\Delta\theta_{ij}^n))S_i^nS_j^n\big), \\ &= Pf_P(\Delta\theta_{ij}^n)S_i^nS_j^n + w_{ij}^{n-1}F(\Delta\theta_{ij}^n), \end{aligned} \tag{1}$$

where $S_i^n = 1$ if cell i is a place cell in environment n and otherwise is zero. We use Eq. 1 iteratively, to solve for w_{ij}^n in terms of the weights for environment $n-2$, $n-3$ and so on, until we arrive at a formulation in terms of the plasticity occurred in environment $n-\eta$. This is precisely Eq.30 from the Methods. In a slight abuse of notation, which however will make the calculation easier to follow, we will write $f_{P,\eta} = f_P(\Delta\theta_{ij}^{n-\eta})$, which indicates that the plasticity function for potentiation should be ordered in the environment with age η, i.e. environment $n-\eta$. Using similar notation (indicating the age and not the exact identity of the memory) for F, C and w we arrive at

$$w_{ij}^n = \big(Pf_{P,\eta}S_i^\eta S_j^\eta + w_{ij}^{\eta+1}F_\eta\big)\cdot\prod_{l=0}^{\eta-1}F_l + P\sum_{k=0}^{\eta-1}f_{P,k}S_i^kS_j^k\prod_{l=0}^{k-1}F_l. \tag{2}$$

We recall that the mean and variance of the weights are written μ_w and σ_w^2, and are given by Eqs.14 and 16 in the Methods. Using these two statistics and Eq.2 we can calculate the correlation of the weight matrix with environment $n-\eta$. Specifically, we calculate the amplitude $a_\eta = 2\big\langle\cos(\Delta\theta_{ij}^{n-\eta}), w_{ij}^n\big\rangle$, where the brackets indicate an average over all neurons. We note that in Eq.2, the functions $f_{P,\eta}$ and F_η both have phases ordered in environment $n-\eta$, while all other terms have orderings which are random and uncorrelated with this space. We also note that we have defined $f_p(\theta) = 1+\cos\theta$ and $f_D(\theta) = 1-\cos\theta$, which means that $F(\theta) = 1-[(P+D)-(D-P)\cos\theta]S_iS_j$. The averages of these functions over neurons are $\big\langle f_p\big\rangle = \big\langle f_D\big\rangle = 1$, and $\big\langle F\big\rangle = 1-s^2(P+D)$, where s is the coding sparseness. We will also need the fact that $\big\langle F^2\big\rangle = 1+s^2\left(\frac{3}{2}(P^2+D^2)+PD-2(P+D)\right)$. These last two relations come about due to the fact that $\big\langle S_iS_j\big\rangle = \big\langle S_i\big\rangle\big\langle S_j\big\rangle = s^2$, i.e. the expected value of S_i is one times the probability of a neuron being active s, and also $\big\langle (S_i)^2\big\rangle = \big\langle S_i\big\rangle = s$.

Therefore

$$\begin{aligned} a_\eta &= 2\Big\langle\cos(\Delta\theta_{ij}^{n-\eta})\cdot\big(Pf_{P,\eta}S_i^\eta S_j^\eta + w_{ij}^{\eta+1}F_\eta\big)\cdot\prod_{l=0}^{\eta-1}F_l\Big\rangle, \\ &= \frac{1}{\pi}\int_{-\pi}^{\pi}\big(P(1+\cos\theta)+\mu_w(1-D-P+(D-P)\cos\theta)\big)\cos\theta d\theta\cdot\big\langle F\big\rangle^\eta, \\ &= \frac{2PD}{P+D}(1-s^2(P+D))^\eta, \end{aligned} \tag{3}$$

where we have used the fact that $\mu_w = P/(P+D)$ and $S_i^\eta = 1$ as we are considering only active place cells in that environment. The amplitude a_η is the amplitude of the first Fourier mode of the spatial connectivity in space η. For the simple plasticity functions considered here it provides a complete characterization of the spatial modulation. Now, the mean synaptic connectivity ordered in space η is

$$M_\eta = \mu_w + a_\eta\cos\big(\Delta\theta^{n-\eta}\big).$$

However, if we consider the connectivity profile for a specific post-synaptic cell i, it will deviate from this mean due to the quenched variability caused by global remapping.

The variance of the connectivity about this *ordered* mean is

$$\begin{aligned} V_\eta &= \left\langle \left(w_{ij}^n - M_\eta \right)^2 \right\rangle \\ &= \left\langle \left(w_{ij}^n - a_\eta \cos\left(\Delta\theta^{n-\eta}\right) \right)^2 \right\rangle - \mu_w^2 \\ &= A_\eta + B_\eta \cos\left(\Delta\theta^{n-\eta}\right) + C_\eta \cos^2\left(\Delta\theta^{n-\eta}\right), \end{aligned} \tag{4}$$

where the brackets now indicate an average over neurons, but carried out for each $\Delta\theta^{n-\eta}$ between neuronal pairs, i.e. there is no averaging over $\Delta\theta^{n-\eta}$. Using equation Eq.2, we find that

$$\begin{aligned} A_\eta \;=\;& \left\langle \left(P + w(1-(P+D)) \right)^2 \prod_{l=0}^{\eta-1} F_l^2 \right\rangle - \mu_w^2 \\ & + 2P \left\langle \left(P + w(1-(P+D)) \right) \prod_{l=0}^{\eta-1} F_l \cdot \sum_{k=0}^{\eta-1} f_{p,k} S_i^k S_j^k \prod_{l=0}^{k-1} F_l \right\rangle \\ & + P^2 \left\langle \sum_{k=0}^{\eta-1} f_{p,k} S_i^k S_j^k \prod_{l=0}^{k-1} F_l \sum_{m=0}^{\eta-1} f_{p,m} S_i^m S_j^m \prod_{r=0}^{m-1} F_r \right\rangle \\ =\;& \left\langle (P^2 + 2Pw(1-P-D) + w^2(1-P-D)^2) \right\rangle \prod_{l=0}^{\eta-1} \left\langle F^2 \right\rangle - \mu_w^2 \\ & + 2P \left\langle (P + w(1-P-D)) \right\rangle \sum_{k=0}^{\eta-1} \left\langle f_{p,k} F_k S_i^k S_j^k \right\rangle \prod_{l=0}^{k-1} \left\langle F^2 \right\rangle \prod_{r=k+1}^{\eta-1} \left\langle F \right\rangle \\ & + P^2 \sum_{k=0}^{\eta-1} \left\langle (f_{p,k})^2 \right\rangle \left\langle (S_i^k)^2 \right\rangle \left\langle (S_j^k)^2 \right\rangle \prod_{l=0}^{k-1} \left\langle F_l^2 \right\rangle \\ & + 2P^2 \sum_{l=1}^{\eta-1} \sum_{k=0}^{l-1} \left\langle f_{p,l} \right\rangle \left\langle f_{p,k} F_k \right\rangle \left\langle S_i^l \right\rangle \left\langle S_j^l \right\rangle \left\langle S_i^k \right\rangle \left\langle S_j^k \right\rangle \prod_{m=0}^{k-1} \left\langle F_m^2 \right\rangle \prod_{r=k+1}^{l-1} \left\langle F_r \right\rangle \\ =\;& \left(P^2 + 2P\mu_w(1-P-D) + \left\langle w^2 \right\rangle (1-P-D)^2 \right) \left\langle F^2 \right\rangle^\eta \\ & + 2P \left(P + \mu_w(1-P-D) \right) \left\langle f_p F \right\rangle s^2 \cdot \frac{\left\langle F \right\rangle^\eta - \left\langle F^2 \right\rangle}{\left\langle F \right\rangle - \left\langle F^2 \right\rangle} \\ & + P^2 s^2 \left\langle f_p^2 \right\rangle \frac{1 - \left\langle F^2 \right\rangle^\eta}{1 - \left\langle F^2 \right\rangle} \\ & + 2P^2 s^4 \frac{\left\langle f_p F \right\rangle}{\left\langle F \right\rangle - \left\langle F^2 \right\rangle} \left(\frac{1 - \left\langle F \right\rangle^\eta}{1 - \left\langle F \right\rangle} - \frac{1 - \left\langle F^2 \right\rangle^\eta}{1 - \left\langle F^2 \right\rangle} \right), \end{aligned} \tag{5}$$

where we have used the fact that $S_k = \sum_{i=0}^{k-1} z^i = \frac{1-z^k}{1-z}$.

$$
\begin{aligned}
B_\eta &= 2\Big\langle \big[\big(P + w(1-P-D)\big)\prod_{l=0}^{\eta-1} F_l + P\sum_{k=0}^{\eta-1} f_{p,k} S_i^k S_j^k \prod_{l=0}^{k-1} F_l\big] \\
&\quad \cdot\big[\big(P - w(P-D)\big)\prod_{l=0}^{\eta-1} - a_0 \langle F\rangle^\eta\big]\Big\rangle \\
&= 2\Big\langle \big(P + w(1-P-D)\big)\big(P - w(P-D)\big)\prod_{l=0}^{\eta-1} F_l^2 \Big\rangle \\
&\quad + 2P\Big\langle \big(P - w(P-D)\big)\sum_{k=0}^{\eta-1} f_{p,k} F_k S_i^k S_j^k \prod_{l=0}^{k-1} F_l^2 \prod_{j=k+1}^{\eta-1} F_j \Big\rangle \\
&\quad - 2a_0\langle F\rangle^\eta \Big(\Big\langle \big(P + w(1-P-D)\big)\prod_{l=0}^{\eta-1} F_l \Big\rangle + P\Big\langle \sum_{k=0}^{\eta-1} f_{p,k} S_i^k S_j^k \prod_{l=0}^{k-1} F_l \Big\rangle\Big) \\
&= 2\big(P^2 + \mu_w P(1-2P) - \langle w^2\rangle (P-D)(1-P-D)\big)\prod_{l=0}^{\eta-1} \langle F\rangle \\
&\quad + 2a_0 s^2 P \sum_{k=0}^{\eta-1} \langle f_p F\rangle \prod_{l=0}^{k-1} \langle F^2\rangle \prod_{j=k+1}^{\eta-1} \langle F\rangle \\
&\quad - 2a_0\mu_w \langle F\rangle^\eta \prod_{l=0}^{\eta-1} \langle F\rangle - 2a_0 s^2 P \langle F\rangle^{\eta-1} \sum_{k=0}^{\eta-1}\prod_{l=0}^{k-1} \langle F\rangle \\
&= 2\big(P^2 + P\mu_w(1-2P) - \langle w^2\rangle (P-D)(1-P-D)\big)\langle F^2\rangle - 2a_0\mu_w \langle F\rangle^{2\eta} \\
&\quad + 2a_0 s^2 P\Big(\langle f_p F\rangle \frac{\langle F\rangle^\eta - \langle F^2\rangle^\eta}{\langle F\rangle - \langle F^2\rangle} - \frac{\langle F\rangle^\eta - \langle F\rangle^{2\eta}}{1 - \langle F\rangle}\Big) \\
&= 2\frac{(P-D)}{(P+D)}\big(P^2 + P\mu_w(1-2P-2D) - \langle w^2\rangle (P+D)(1-P-D)\big)\langle F^2\rangle^\eta \\
&\quad + 2a_0\mu_w\big(\langle F^2\rangle^\eta - \langle F\rangle^{2\eta}\big) \\
&\quad + 2a_0 s^2 P\Big(\langle f_p F\rangle \frac{\langle F\rangle^\eta - \langle F^2\rangle^\eta}{\langle F\rangle - \langle F^2\rangle} - \frac{\langle F\rangle^\eta - \langle F\rangle^{2\eta}}{1 - \langle F\rangle}\Big),
\end{aligned} \tag{6}
$$

where we have used the fact that $P - \mu_w(P-D) = 2PD/(P+D) = a_0$. Finally we have

$$
\begin{aligned}
C_\eta &= \Big\langle \big((P - w(P-D))\prod_{l=0}^{\eta-1} F_l - a_0\langle F\rangle^\eta\big)^2 \Big\rangle \\
&= \Big\langle (P^2 - 2wP(P-D) + w^2(P-D)^2 \prod_{l=0}^{\eta-1} F_l^2 \Big\rangle \\
&\quad - 2a_0\langle F\rangle^\eta \Big\langle (P - w(P-D))\prod_{l=0}^{\eta-1} F_l \Big\rangle + a_0^2\langle F\rangle^{2\eta} \\
&= \big(P^2 - 2\mu_w P(P-D) + \langle w^2\rangle (P-D)^2\big)\langle F^2\rangle^\eta \\
&\quad - 2a_0(P - \mu_w(P-D))\langle F\rangle^{2\eta} + a_0^2\langle F\rangle^{2\eta} \\
&= \big(\frac{P-D}{P+D}\big)^2\big(P^2 - 2P\mu_w(P+D) + \langle w^2\rangle (P+D)^2\big) \\
&\quad + a_0^2\big(\langle F^2\rangle^\eta - \langle F\rangle^{2\eta}\big).
\end{aligned} \tag{7}
$$
