# Supplementary material for: Rapid memory encoding in a recurrent network model with behavioral time scale synaptic plasticity
Source: PLoS Comput Biol. 2023 Aug 25;19(8):e1011139. doi: 10.1371/journal.pcbi.1011139 (PMC10484462; doi:10.1371/journal.pcbi.1011139)
Supplement: S2 Appendix — (PDF) [file pcbi.1011139.s002.pdf]

Calculation of the Spatial Fourier Spectrum for Quenched White Noise

The quenched variability which arises through the global remapping of place cells when recurrent weights are shaped by BTSP, takes the form $\Delta W(\theta) = \sqrt{V(\theta)}z(\theta)$, where $V(\theta) = A + B\cos\theta + C\cos^2\theta$ is the variance calculated in the previous section, and z is a zero-mean Gaussian random variable with unit variance. We understand θ here to be the phase difference between the centroid of the place fields of a pair of neurons. We write $\Delta W(\theta)$ in terms of the finite Fourier series

$$\Delta W(\theta) = 2\sum_{j=1}^{N}\alpha_j\cos(j\theta) - 2\sum_{j=1}^{N}\beta_j\sin(j\theta). \tag{1}$$

The coefficients α_j and β_j are zero-mean Gaussian random variables with variances and covariances which must be determined self-consistently. Specifically, we average both sides over the variability at each position θ. This means an average over neurons, or equivalently, an average over the distribution of the αjs and β_js. Specifically we have $V(\theta) = \langle\Delta W(\theta)^2\rangle$, where

$$\begin{aligned} V(\theta) &= 2\sum_{j=1}^{N}\sum_{l=1}^{N}\big(\langle\alpha_j\alpha_l\rangle + \langle\beta_j\beta_l\rangle\big)\cos((j-l)\theta) + 2\sum_{j=1}^{N}\sum_{l=1}^{N}\big(\langle\alpha_j\alpha_l\rangle - \langle\beta_j\beta_l\rangle\big)\cos((j+l)\theta) \\ &\quad + 4\sum_{j=1}^{N}\sum_{l=1}^{N}\big(\langle\alpha_j\beta l\rangle\sin((j-l)\theta) - \langle\alpha_l\beta j\rangle\sin((j+l)\theta)\big). \end{aligned} \tag{2}$$

We then calculate the Fourier coefficients explicitly for the only non-zero modes. For this, we rewrite the variance as $V(\theta) = A + \frac{C}{2} + B\cos(\theta) + \frac{C}{2}\cos(2\theta)$. We now make use of Parseval's theorem, which roughly speaking states that the power in the signal is conserved in Fourier space. This leads to the following relations

$$\frac{1}{2}\int_{-\pi}^{\pi}V(\theta)d\theta = A + \frac{C}{2} = \sum_{j=1}^{N}\big(\langle\alpha_j^2\rangle + \langle\beta_j^2\rangle\big), \tag{3}$$

$$\frac{1}{2}\int_{-\pi}^{\pi}V(\theta)\cos\theta d\theta = B = \sum_{j=1}^{N-1}\big(\langle\alpha_j\alpha_{j+1}\rangle + \langle\beta_j\beta_{j+1}\rangle\big), \tag{4}$$

$$\frac{1}{2}\int_{-\pi}^{\pi}V(\theta)\cos 2\theta d\theta = \frac{C}{2} = \sum_{j=1}^{N-2}\big(\langle\alpha_j\alpha_{j+2}\rangle + \langle\beta_j\beta_{j+2}\rangle\big) + \langle\alpha_1^2\rangle - \langle\beta_1^2\rangle. \tag{5}$$

Eqs.3-5 provide only three constraints for a large number of unknowns, of order N. However, we can use the fact that the power spectrum of a white noise process is flat, meaning that the power is spread evenly amongst all modes. This leads to Eqs.37-40 of the main text. Note that the variance of the coefficients of the first mode is a special case because we have two constraints

$$\begin{aligned} \langle\alpha_1^2\rangle + \langle\beta_1^2\rangle &= \frac{1}{2N}\big(A + \frac{C}{2}\big), \\ \langle\alpha_1^2\rangle - \langle\beta_1^2\rangle &= \frac{C}{4N}, \end{aligned}$$

where the second relation arises due to the spatial inhomogeneity of the quenched variability. A comparison of this theory with numerical simulation of a quenched, Gaussian white-noise process is shown in Fig 1 and 2.

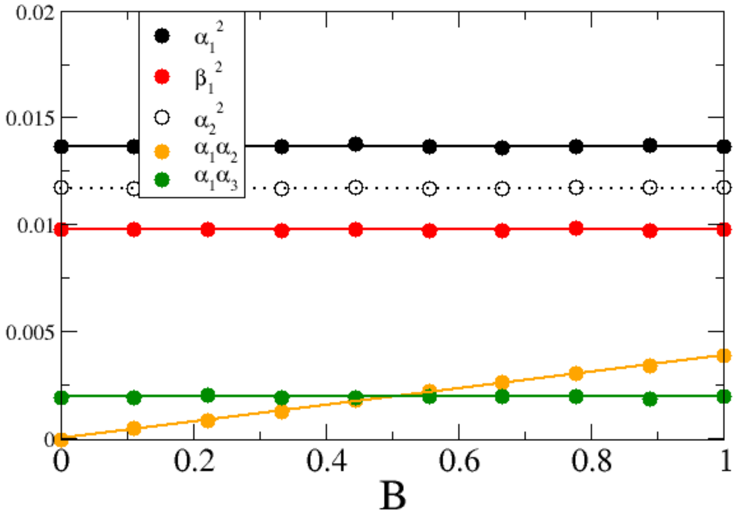

Fig 1. Comparison of the theory (lines) with numerical simulations for different values of B. Parameters are: A = 1, C = 1, N = 64. Symbols are averages of 10,000 realizations of a quenched, Gaussian white noise process on a ring with variance V_θ.

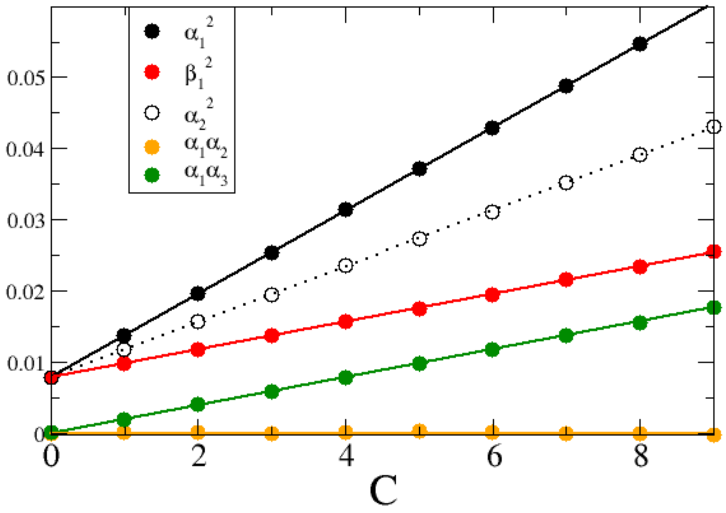

Fig 2. Comparison of the theory (lines) with numerical simulations for different values of C. Parameters are: A = 1, C = 0, N = 64. Symbols are averages of 10,000 realizations of a quenched, Gaussian white noise process on a ring with variance V_θ.

Here we can see the role of N, the number of encoded positions around the ring. Although the power (variance) of the quenched variability is entirely independent of N, the power in each Fourier mode decreases as $1/N$. Therefore, we can expect that in the vicinity of an instability to a spatially modulation mode, the value of N will be important. In fact, in the limit as $N \to \infty$ the noiseless case is recovered, even though the total power in the quenched variability is unaffected. On the other hand, for finite N any spatial bifurcation will be shifted.

In the case of the Turing bifurcation, we can calculate this effect analytically. Specifically, the connectivity profile for a cell i is $W(\theta_i - \theta_j) = W_0 + W_1 \cos(\theta_i - \theta_j) + \sqrt{V(\theta_i - \theta_j)} z(\theta_i - \theta_j)$. It is important to note that the value of z is different for each neuronal pair (i, j). To know how the bifurcation is shifted, We should extract the component of the variability which is commensurate with the cosine mode. This has the form $R \cos \theta - \psi$, where $R = 2\sqrt{\alpha^2 + \beta^2}$ and $\psi = \tan^{-1}(\beta/\alpha)$. The amplitude and phase R and ψ are therefore also random variables. We can calculate the mean amplitude by integrating over the distributions of α and β

$$\begin{aligned}
\langle R \rangle &= \frac{1}{\pi\sigma^2} \int_{-\infty}^{\infty} \int_{-\infty}^{\infty} d\bar{x} d\bar{y} \sqrt{\bar{x}^2 + \bar{y}^2} e^{-\frac{\bar{x}^2}{2\sigma^2}} e^{-\frac{\bar{y}^2}{2\sigma^2}} \\
&= \frac{2\sqrt{2}\sigma}{\pi} \int_{-\infty}^{\infty} \int_{-\infty}^{\infty} dxdy \sqrt{x^2 + y^2} e^{-x^2 - y^2} \\
&= 4\sqrt{2}\sigma \int_{0}^{\infty} dr \cdot r^2 e^{-r^2} \\
&= 4\sqrt{2}\sigma \Big[\frac{\sqrt{\pi}}{4} \text{erf}(r) - \frac{1}{2} e^{-r^2} \Big]_0^{\infty} \\
&= \sqrt{2\pi}\sigma,
\end{aligned} \tag{6}$$

where $\sigma^2 = \frac{1}{N}\left(A + \frac{C}{2}\right)$, and the last integral is obtained by assuming polar coordinates, for which $dxdy \to rdrd\theta$. To solve the integral analytically we have assumed that the variances of α_j and β_j are the same, which is not the case for $j = 1$ unless $C = 0$. In that case the integral can be solved numerically.

On average then, the quenched variability will contribute an amplitude $\langle R \rangle$ to the connectivity, however the phase is also important. If $\psi = 0$ then the quenched variability will add to W_1 and make an instability more likely. However, if $\psi = \pi$ then the quenched variability will have the opposite effect and make an instability less likely. Given that there is a distinct phase ψ for each neuron in the network, if N is learge enough we can expect that for some of them $\psi \sim 0$. The quenched variability therefore always makes the instability more likely, shifting the bifurcation to lower values of W_1, although the emergent bump will be biased to certain locations. Note that the fact that $\alpha_1 > \beta_1$ due to the spatial inhomogeneity of the noise through the coefficient C means that ψ will, in fact, be biased to values near 0, reducing the effect of so-called "hot spots". This effect requires further study.
