# Supplementary figures and images for: Rapid memory encoding in a recurrent network model with behavioral time scale synaptic plasticity
Source: PLoS Comput Biol. 2023 Aug 25;19(8):e1011139. doi: 10.1371/journal.pcbi.1011139 (PMC10484462; doi:10.1371/journal.pcbi.1011139)

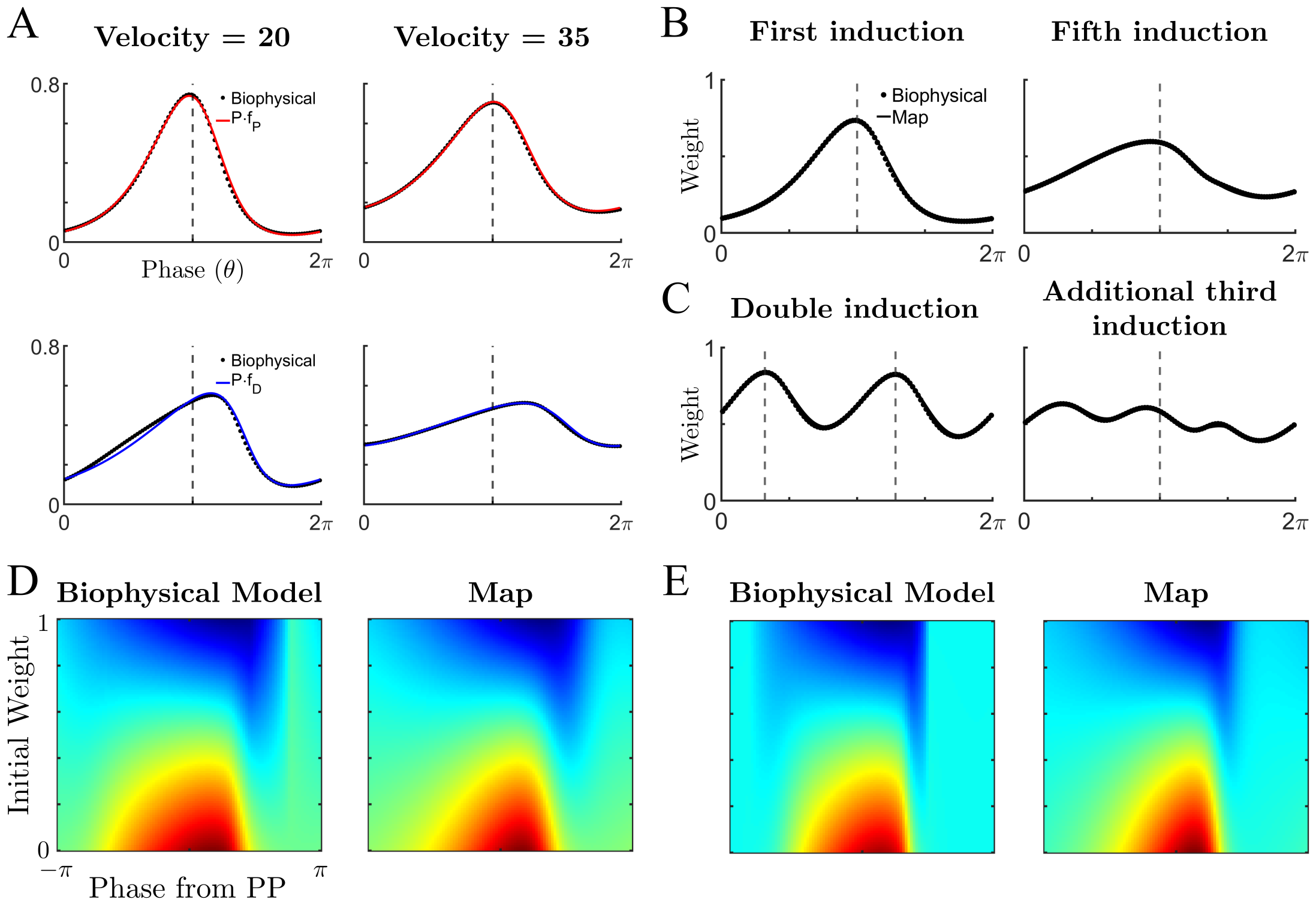

Supplement: S1 Fig — (A) Potentiation (top) and depression (bottom) plasticity functions for different velocities. (B-C) v = 25cm/s. (B) Synaptic weights after a single induction (left) and after five inductions at the same position on consecutive trials (right). (C) The results of two inductions at distinct locations on the same trial, and the subsequent change in weights after a third induction on the second trial (right). (D-E) The 1D map quantitatively captures the degree of plasticity inferred from the biophysical model with different parameter set. Parameters: (A) Velocity = 20: P = 2.625, μP = 0.75, σP = 1.825, λP = −1.55, νP = 5, D = 2.8, μD = 1.875, σD = 3.45, λD = −4.55, and νD = 5; velocity = 35: P = 2, μP = 0.6, σP = 1.42, λP = −1.625, νP = 2, D = 2.15, μD = 1.54, σD = 3.85, λD = −5.75, and νD = 3. (B-C) Model parameters are the same as Fig 1. In panel B, xPPi=93.5 cm, middle of the track, for i ∈ {1, 2, 3, 4, 5}. In panel C, xPP1=30,120 cm and xPP2=93.5 cm. (D-E) Setup of simulation: v = 35cm/s, L = 187cm, xpp = 93.7 cm. Parameters of biophysical model for panel D (E) are: τElig = 0.98(0.8), τIS = 0.64(0.5), α+ = 0.19(0.2), β+ = 23.82(30), α− = 0.05(0.1), β− = 730.64(1000), k+ = 0.8(0.8), k− = 0.44(0.4). The corresponding parameters of 1D map are νP = 6(5), μP = 0.95(0.8), σP = 1.45(1.35), λP = −4.5(−6), P = 2(1.6), νD = 5(5), μD = 1.6(1.1), σD = 2.5(1.6), λD = −6(−7), D = 2.65(1.2). (TIF) [file pcbi.1011139.s003.tif]

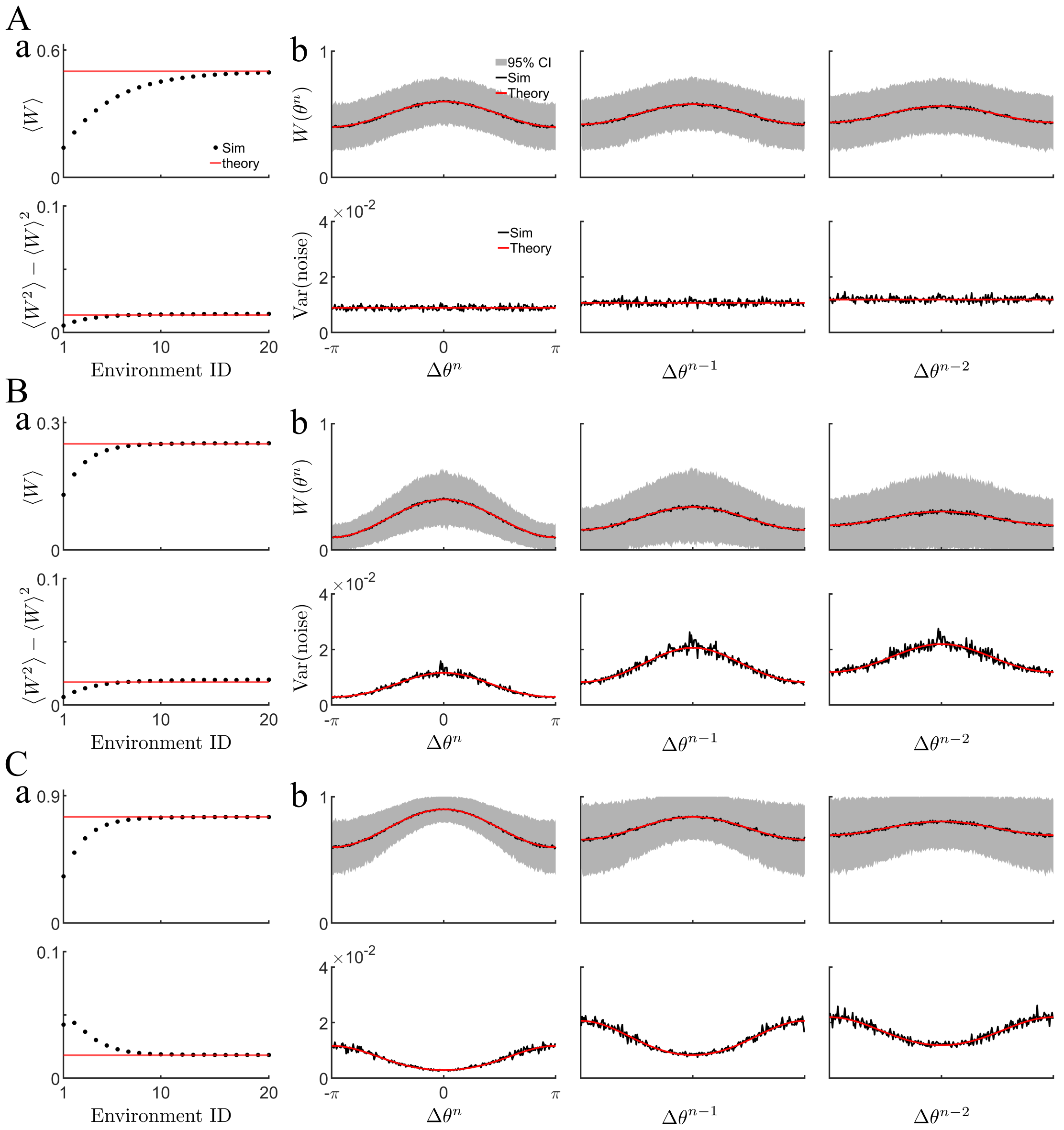

Supplement: S2 Fig — (A-C) a. Global statistics, average (top) and variance (bottom), as function of number of explored environments. b. Spatial statistics of weight matrix at steady state: mean curve with 95% confidence interval (top) and variance of noise (bottom) as function of phase difference. Parameters: (A) P = D = 0.1; (B) P = 0.1 and D = 0.3; (C) P = 0.3 and D = 0.1. (TIF) [file pcbi.1011139.s004.tif]

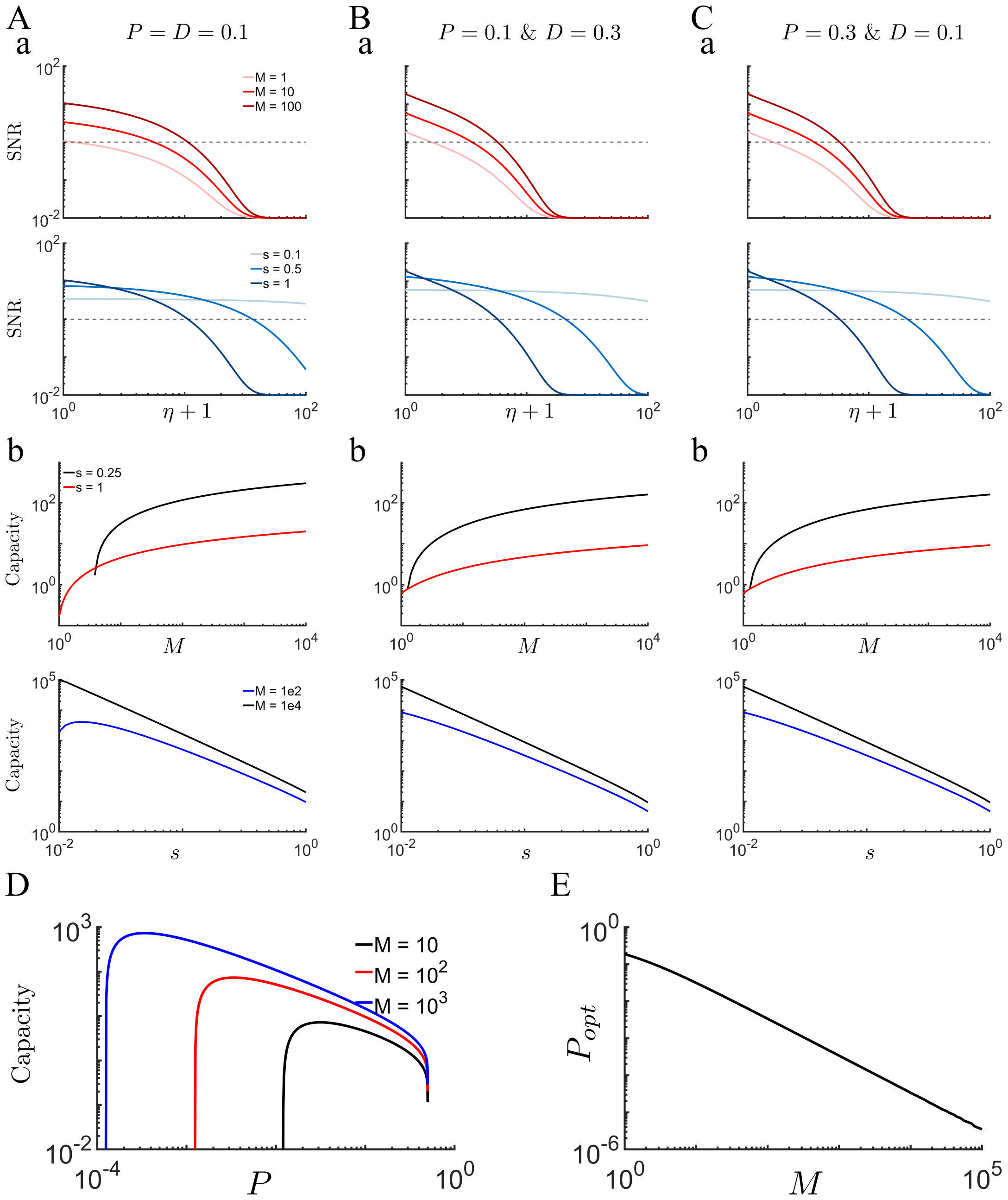

Supplement: S3 Fig — (A-C) a. Signal-to-noise ratio as function of number of past explored environments by fixing either sparsity (top) or network size (bottom). Dashed line indicates the inferior threshold that no memory trace lower than this value. b. Memory capacity of network as function of network size (top) or sparsity (bottom). (D-E) P = D. (D) The memory capacity of system as function of P. (E) The optimal value of P that maximize the capacity of system as function of population size M. (TIF) [file pcbi.1011139.s005.tif]

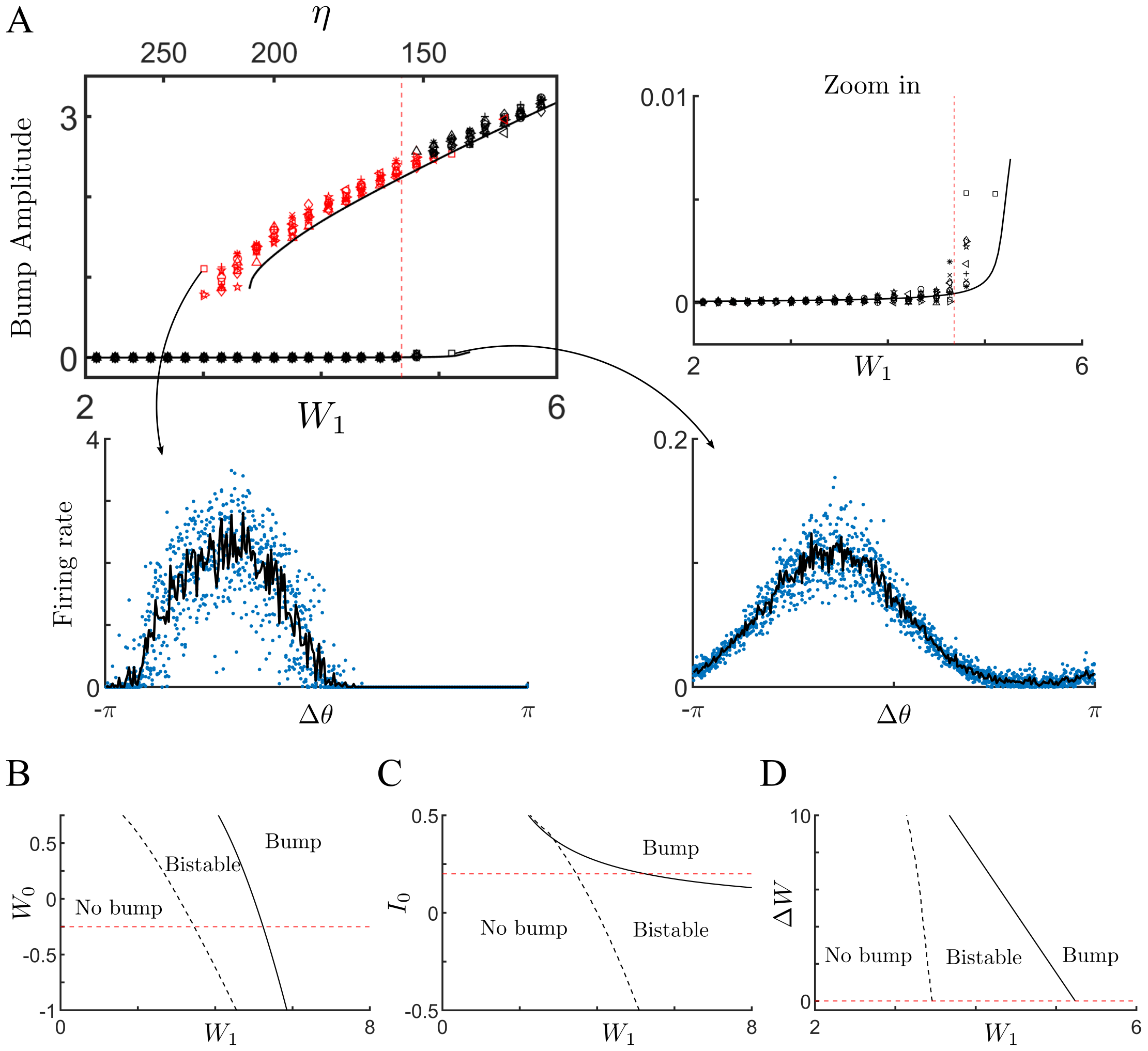

Supplement: S4 Fig — (A) Left top: Zoomed plot of the Fig 4C showing bump amplitude in individual fashion. Right top: Detail of diagram highlighting the emergence of a small amplitude, supercritical Turing bifurcation. Bottom: sample profile of large and small bump amplitude. (B-D): Phase diagrams of the ring model without noise. The solid line indicates the analytical expression of the Turing bifurcation, while the dashed line indicates the saddle-node of bump solution. Red dashed line represents the same network setup across phase diagrams. (TIF) [file pcbi.1011139.s006.tif]

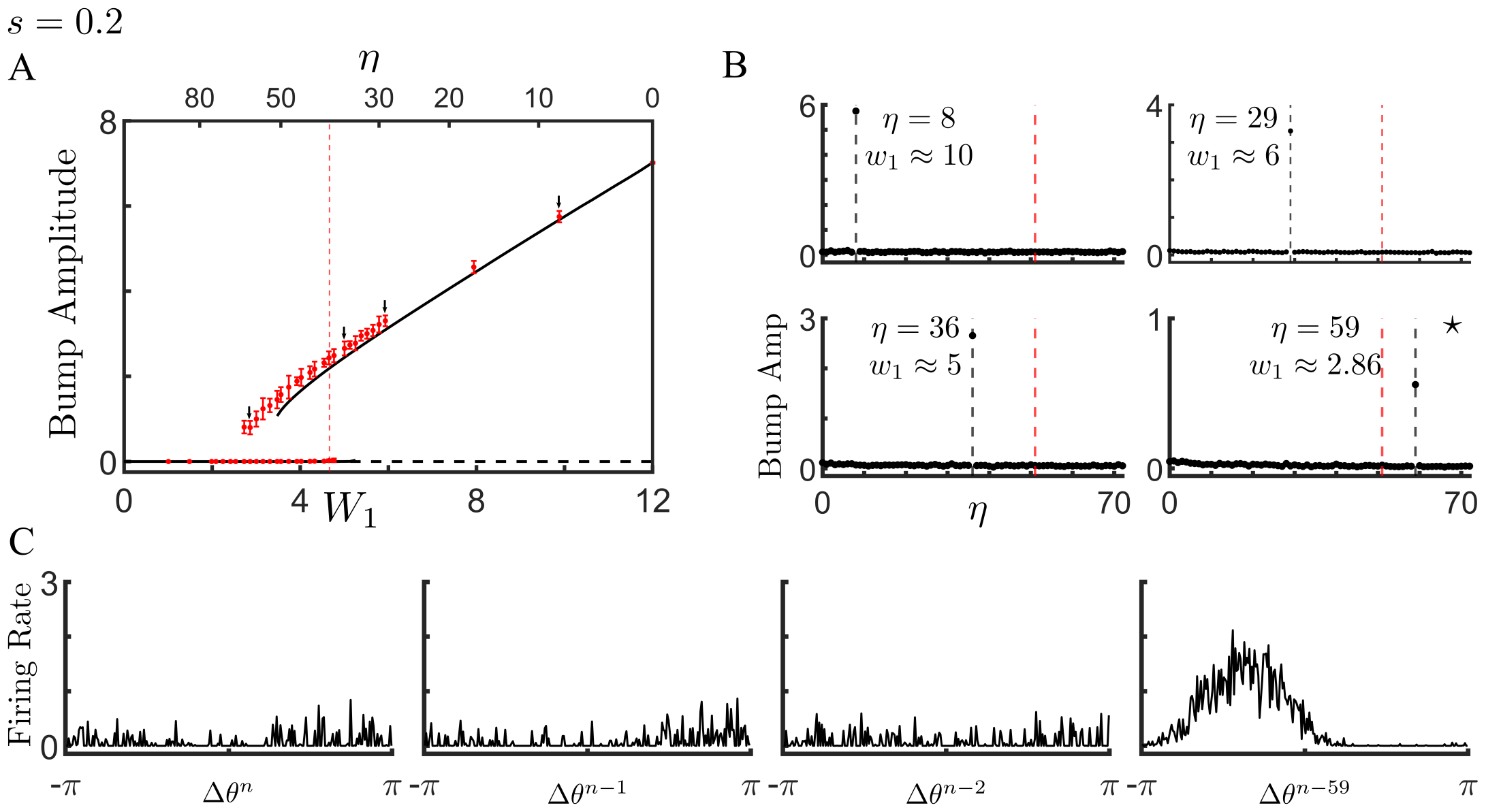

Supplement: S5 Fig — (TIF) [file pcbi.1011139.s007.tif]
