# Supplementary material for: Rapid memory encoding in a recurrent network model with behavioral time scale synaptic plasticity
Source: PLoS Comput Biol. 2023 Aug 25;19(8):e1011139. doi: 10.1371/journal.pcbi.1011139 (PMC10484462; doi:10.1371/journal.pcbi.1011139)
Supplement: Attachment — Submitted filename: review_PLOS_00687.pdf [file pcbi.1011139.s008.pdf]

In this manuscript, “Rapid memory encoding in a recurrent network model with behavioral time scale synaptic plasticity”, the authors provide a strong mathematical analysis of memory encoding and recall in a recurrent neural network with a BTSP-like learning rule. However, I am concerned that the assumption of temporally symmetric plasticity regarding BTSP diverges too far from what is suggested by the existing literature. I am also concerned that the work does not adequately compare its results to those of classic memory models. Further, given the experimental relevance of BTSP, I think the work suffers from a lack of explicit testable predictions and falsifiability. I have listed my major concerns in bold. I would feel comfortable recommending the paper only if the authors make significant revisions which address adequately these major concerns.

Text:

Line 11, recurring: What do you mean by quenched variability here? quenched in the neuroscience sense (lowered neural variability due to attention to a task, for example) or quenched in the statistical mechanics sense? Need to describe exactly how and why the variability is “quenched”.

Line 50, recurring: should briefly describe what you mean by "map" here

Line 67: What are the experimental predictions of your model/analysis? Is your model (or the results of your analysis) falsifiable or testable? Edit: I acknowledge that the abstract mentions the following: “We show that the calculated memory trace can be used to predict the emergence and stability of bump attractors in a high dimensional neural network model endowed with BTSP”, but I mean “predict” as in experimental predictions from your analysis, not predictions of simulated network behavior.

Line 93: “It does not explicitly depend on continuous time” – indeed, but it does implicitly since it depends on phase (in units of either space or time). I don’t see how this is categorically different than the learning rules of Milstein et al. or the analysis of Cone and Shouval which looks at offline updates and fixed points of the rule (which also only depend on phase) …

Edit: this point has made more sense after reading through the methods, but I still am cautious. I think when describing a “map” you should explicitly define it the first time you use it: that you are replacing phase dependent plasticity with phase dependent approximations of plasticity, which allows for tractable analysis.

Line 119, recurring: should describe how this rule is "BTSP-like" once you have approximated spatially dependent plasticity functions. In theory f_P and f_D could be replaced by any function, not just those in panel 1f. There are certainly choices of f_P and f_D for which I wouldn't consider this rule to be "BTSP". Later, you approximate f_P and f_D as symmetric, yet the functions derived from the Milstein et al BTSP rule are asymmetric. I understand that this assumption was made for analytical tractability, but as you mention, the asymmetry can lead to dynamic attractors (as opposed to the bump attractors you examine in this work). Spalla et al. 2021 analyzes the memory capacity of dynamic attractor networks. Can you leverage the findings of this previous work to include a section (with a figure from new simulations) on the memory capacity of your network with the asymmetric plasticity rule?

Line 191, recurring: what is a "temporally symmetric BTSP rule"? equation 2, with undefined f_p and f_D, is just a weight dependent plasticity rule, no? Could you not fit f_P and f_D to, for example: an STDP kernel (or any other LTP/LTD kernel) and get the same results?

The part that makes it "BTSP" is the fitting of f_P and f_D to data related to BTSP. This is an important point I think needs to be addressed. How much do your results critically depend on the identifying factors of BTSP (asymmetric plasticity kernel, weight dependence, long timescale plasticity, one-shot learning, translocation of fields, etc.), and can you show this?

Line 246, recurring: should explicitly compare to classical results and show this alongside your model results in a figure. For example, how does your model's memory trace compare to that of a Hopfield net subjected to the same environment-specific remapping?

Line 417: Large compared to what? A recurrent network endowed with Hebb or STDP rules? Then show this explicitly in a figure, and/or compare the expressions for capacity side by side so it is obvious.

Line 453: this point is still unclear. If this is a main takeaway of the work, one of the figures should explicitly show this (bump attractors in certain regions do not form without this variability, and with variability, they do.) I think that this is shown in Fig 5a and 5d, but if that is the case, the language should be explicit, and refer back to the figure. E.g. "Perhaps surprisingly, the effect of this variability is to increase the memory capacity by allowing for the formation of mixed-state bump attractors for large eta/small W1…"

Line 474: see again note for line 191 – results showing BTSP have found the temporally asymmetry to be a defining characteristic. Need to explain why "temporally symmetric BTSP" is still BTSP.

Line 481: as in, the time between environments, or the time between plateau events in a single environment?

Line 564: repeat comment, but could all this same analysis be applied to a recurrent network encoding memories via other plasticity rules?

<u>Formatting/Presentation:</u>

In all figures: increase font size and line width where possible to improve readability.

Fig 1: make use of colors consistent, indicate what colors mean in each panel. Also, panel E is a key part of the paper, but it is hard to understand. This didn't make sense to me prior to the methods. I think it is possible to convey using a more descriptive figure.

Fig 4:

panel a) write out initial condition rather than "I.C."

panel c and d) describe the vertical red and black dotted lines (panel D), and both vertical red horizontal black dotted lines in panel C. Figure caption for panel C refers to "dotted line" but unclear which one it is referring to.

Panel d) Since eta goes to 250, hard to distinguish the dots from each other - this is much more legible in figure 5b. Maybe only plot every 10 eta? star is confusing since it is at the same height and is of comparable size/same color as the elevated dot.

Panel e) clarifying that this is environment n-30 would help readability (both in panel and in figure caption), even though it is implied by the star in D.

Panel e) describe what the dots mean

Panel f) show instances of the network in each of the three regimes (in the style of panel a). Also, if all three regimes exist for w0>=0, why set it to be negative? Doesn't this add an extra assumption of inhibition which is unnecessary?

Fig 5: Unclear what "measured with different orderings" means. Do you mean measured in different environments (which have their unique ordering)? If so, pick one naming convention ("environment" is probably the most intuitive) and stick to it. If not, more description is needed to distinguish "measured with different orderings" and "measured in different environments"

Fig 6: please show statistical significance, unclear to what degree the network actually outperforms the ring model approximation. Define what "optimal" means in "scales optimally" and compare panel B to classical memory networks.

A few typos:

In abstract: "simplified to simple map" -> "simplified to a simple map"

Eq. 2: missing closing parenthesis for (1-W)

Line 638: subscript missing for a_eta.

Line 648: "memory" misspelled
