# Supplementary material for: Rapid memory encoding in a recurrent network model with behavioral time scale synaptic plasticity
Source: PLoS Comput Biol. 2023 Aug 25;19(8):e1011139. doi: 10.1371/journal.pcbi.1011139 (PMC10484462; doi:10.1371/journal.pcbi.1011139)
Supplement: Attachment — Submitted filename: reply_to_reviewers_YeRoxin2023.pdf [file pcbi.1011139.s009.pdf]

Reply to Reviewer 1

The reviewer's main concern regards the symmetric nature of the BTSP rule which we use, and the lack of comparison with experimental results. When we carried out the research, the choice of a symmetric rule was made in order to allow for the formation of stable bump attractors, which have been hypothesized to underlie the storage of spatial maps in recurrent networks in CA3. This symmetry itself can be considered a strong prediction of our model. We are therefore encouraged by the recent (30th of May, 2023) publication of a preprint from the laboratory of Jeff Magee, showing that BTSP in CA3 is indeed symmetric https://www.biorxiv.org/content/10.1101/2023.05.30.542781v1. We address the reviewer's other concerns and comments in detail in what follows.

Line 11, recurring: What do you mean by quenched variability here? quenched in the neuroscience sense (lowered neural variability due to attention to a task, for example) or quenched in the statistical mechanics sense? Need to describe exactly how and why the variability is "quenched".

We mean quenched variability in the statistical mechanical sense. We now write that it is "quenched variability in the network connectivity" to make this clearer.

Line 50, recurring: should briefly describe what you mean by "map" here

We use "map" here in its strict mathematical sense from the field of dynamical systems. We have altered the text to make this clearer. We now write, "Namely, the state of any given synapse in the weight matrix can be expressed as the same weight at a previous time, plus a change due to the plasticity process."

Line 67: What are the experimental predictions of your model/analysis? Is your model (or the results of your analysis) falsifiable or testable? Edit: I acknowledge that the abstract mentions the following: "We show that the calculated memory trace can be used to predict the emergence and stability of bump attractors in a high dimensional neural network model endowed with BTSP", but I mean "predict" as in experimental predictions from your analysis, not predictions of simulated network behavior.

This is a difficult criticism to address in a straightfoward way. We would first make the point that our model provides quantitative fits to the biophysical model from Milstein et al, which in turn reproduces the experimental findings on BTSP in CA1 (see our Fig.1). In this sense, our model of BTSP is clearly consistent with currently published work on BTSP. Our approach was to derive a model which was consistent with the known properties of BTSP, and then study how it behaves in a recurrent network. For this case we used the same model, but chose the plasticity functions f_p and f_d to be symmetric. The choice of symmetric functions leads to spatially symmetric recurrent coupling, which can support stationary bump attractors. Our specific goal was to perform a

detailed analysis of such a network and calculate the memory storage capacity, something which is not feasible with the more detailed model from Milstein et al. Therefore, we sought to answer the question, "What are the memory storage properties of a recurrent network endowed with BTSP?" Such a study makes very clear predictions about how the capacity scales with network parameters including coding sparseness and network size, but it is hard to imagine how such things can be tested using current experimental techniques. This challenge is not unique to our work, but is shared by a large and growing body of computational studies dating back to Hopfield's seminal work. A more recent example of this approach is (Pereira and Brunel, Neuron 2018) in which the authors study how a plasticity rule inferred from data in monkey inferior temporal cortex (a previous publication of theirs) allows for the storage of large numbers of attractors in a recurrent network. In summary, we believe that taking a known plasticity rule, and extrapolating it to cases which are of direct relevance for animal and human cognition, is a valid approach, even if the results are not currently directly testable.

As a second point, we note that recent in-vivo electrophysiology work in head-fixed mice has shown that BTSP is symmetric in CA3, in contrast with CA1, where it is asymmetric (Li et al, biorxiv 2023.05.30.542781). This can be seen as a vindication of our choice of symmetric plasticity functions in our work.

Line 93: "It does not explicitly depend on continuous time" – indeed, but it does implicitly since it depends on phase (in units of either space or time). I don't see how this is categorically different than the learning rules of Milstein et al. or the analysis of Cone and Shouval which looks at offline updates and fixed points of the rule (which also only depend on phase) ... Edit: this point has made more sense after reading through the methods, but I still am cautious. I think when describing a "map" you should explicitly define it the first time you use it: that you are replacing phase dependent plasticity with phase dependent approximations of plasticity, which allows for tractable analysis.

We absolutely agree that our reduced model is an approximation of the plasticity rule from Milstein et al. But we are not sure that "replacing phase dependent plasticity with phase dependent approximations of plasticity" is an entirely accurate description. We believe it is correct to say that we are replacing a time-dependent plasticity rule on a linear track with an approximation that only depends on phase along that track. In fact, we feel strongly that understanding this approximation is important, which is why we added panel E to Figure 1. We believe this panel shows explicitly precisely the transformation we are using to convert a time-dependent process into a phase-dependent process.

Line 119, recurring: should describe how this rule is "BTSP-like" once you have approximated spatially dependent plasticity functions. In theory f_P and f_D could be replaced by any function, not just those in panel 1f. There are certainly choices of f_P and f_D for which I wouldn't consider this rule to be

"BTSP". Later, you approximate f_P and f_D as symmetric, yet the functions derived from the Milstein et al BTSP rule are asymmetric. I understand that this assumption was made for analytical tractability, but as you mention, the asymmetry can lead to dynamic attractors (as opposed to the bump attractors you examine in this work). Spalla et al. 2021 analyzes the memory capacity of dynamic attractor networks. Can you leverage the findings of this previous work to include a section (with a figure from new simulations) on the memory capacity of your network with the asymmetric plasticity rule?

As mentioned above, it turns out that BTSP in CA3 is symmetric. Perhaps this finding already addresses the reviewer's concern. In any case we would argue that the exact shape of the BTSP kernel is not what makes it BTSP, just as there are STDP kernels of many shapes. From a mechanistic perspective, what makes BTSP qualitatively distinct from other forms of plasticity are: 1 - Its long time scale (hence the name *behavioral time-scale* plasticity), and 2 - The postsynaptic instructive signal, or plateau potential. These two properties are what make BTSP capable of rewiring a large number of presynaptic inputs to a cell over a single traversal of a track, thereby generating a new place cell in a matter of seconds. As an aside, we agree with the reviewer that for some choices of f_P and f_D the rule should not be considered BTSP, namely whenever the time-scale is not behaviorally relevant, e.g. too fast.

It is not the case that we chose the rule to be symmetric for analytical tractability. In fact, our analysis of the plasticity is valid for any functions f_D and f_P, symmetric or otherwise, see Eqs.16-23. Rather, and as stated in the text lines 191-192, we chose a symmetric rule in order to encode stationary bump attractors, which have long been hypothesized to underlie spatial memory in CA3 of rodents. Thank you for the reference (Spalla et al 2021). As mentioned above, our analysis takes into account any arbitrary plasticity functions. Therefore, analyzing asymmetric functions would be straightforward in terms of methodology, although it would constitute a major research effort. Essentially we would need to calculate the amplitude of the asymmetric component for each environment, and then add this to the connectivity in the ring model. This would lead to a Turing-Hopf bifurcation to waves rather than a Turing bifurcation to stationary bumps. We have studied this scenario previously with STDP (Theodoni et al, eLife 2018) for a small number of environments. If we had to guess, we would say that the scaling properties of the network for the storage of waves should be similar to that of bumps, but of course we cannot really know without carrying out the analysis and simulations. In any case, given that BTSP is symmetric in CA3 it seems we have studied the more relevant scenario.

Line 191, recurring: what is a "temporally symmetric BTSP rule"? equation 2, with undefined f_p and f_D, is just a weight dependent plasticity rule, no? Could you not fit f_P and f_D to, for example: an STDP kernel (or any other LTP/LTD kernel) and get the same results? The part that makes it "BTSP" is the fitting of f_P and f_D to data related to BTSP. This is an important point I

think needs to be addressed. How much do your results critically depend on the identifying factors of BTSP (asymmetric plasticity kernel, weight dependence, long timescale plasticity, one-shot learning, translocation of fields, etc.), and can you show this?

We do not agree with the reviewer that the defining characteristic of a BTSP rule is the asymmetry, but rather the two features we listed in our response to the previous point. In any case, BTSP can clearly be symmetric, as evidenced by the recent work from the Magee lab. We now cite that preprint in our manuscript. The question of whether another form of plasticity could lead to a similar network structure is an interesting one. STDP rules require many pre-post pairings and hence traversals of the track. We are not aware of any work with STDP in recurrent networks which can directly address the question of storage capacity of bump attractors; this would entail a completely separate study. The importance of the time-scale and one-shot nature of BTSP are absolutely key in our work, as it is what ensures that network-wide inputs to a post-synaptic cell can all be updated according to our map. If the time-scale were very short, as for STDP, and the learning not one-shot, then we could not use a map, but would need to maintain the time-dependence explicitly. The weight dependence is also crucial, as it ensures that synapses remain bounded, leading to the palimpsest, or overwriting property of the network, which is the hallmark of synapse-based memory systems (see Fusi, Oxford Handbook of Human Memory, our reference [21]).

Line 246, recurring: should explicitly compare to classical results and show this alongside your model results in a figure. For example, how does your model's memory trace compare to that of a Hopfield net subjected to the same environment-specific remapping?

We are aware of only one study that specifically looks at the memory capacity in recurrent networks for bump attractors (Battaglia and Treves, PRE 1998). There, the authors consider a Hopfield-like network. Therefore, there is no learning rule, but rather a prescription for how the synaptic weights are correlated with the desired patterns. In that paper, in the sparse coding limit the memory capacity scales as $1/(s \ln s)$ where s is the sparseness (Eq.67 in that paper), whereas here it scales as $\ln s/s^2$ (our Eq.6). Therefore, it appears that the scaling achieved with BTSP and bounded synapses is better than in a Hopfield net. However, our goal here is not to make a comprehensive review of how different models scale with network parameters. Our focus is on the role of BTSP in recurrent networks. We do cite all of the relevant classical results in [15-24], and provide context in lines 241-251. We are open to citing any papers we might have overlooked, of course.

Line 417: Large compared to what? A recurrent network endowed with Hebb or STDP rules? Then show this explicitly in a figure, and/or compare the expressions for capacity side by side so it is obvious.

We have shown, analytically and numerically, that the number of attractors

scales as the square of the number of neurons encoding each position of the track in the sparse-coding limit. This scaling matches the theoretically achievable upper bound for recurrent networks, see e.g. (Fusi, Oxford Handbook of Human Memory 2021). We believe such a scaling justifies the use of the word large in describing the number of attractors. We agree that a comparison of the memory capacity we find with BTSP to that found with other plasticity rules would be valuable. However, we are not aware of any work with Hebbian or STDP rules which studies the storage capacity of bump attractors in recurrent networks. We ourselves have used an STDP rule to store a small number of bump attractors (Theodoni et al 2018 eLife) but the simulation times are prohibitive for a statistical study needed to pin down the scaling of capacity with system parameters. There is also no analytical theory for calculating this capacity as far as we are aware. In any case, our main focus here is not a comprehensive comparison of different plasticity rules, but rather a study of the memory storage properties of recurrent networks with BTSP.

Line 453: this point is still unclear. If this is a main takeaway of the work, one of the figures should explicitly show this (bump attractors in certain regions do not form without this variability, and with variability, they do.) I think that this is shown in Fig 5a and 5d, but if that is the case, the language should be explicit, and refer back to the figure. E.g. "Perhaps surprisingly, the effect of this variability is to increase the memory capacity by allowing for the formation of mixed-state bump attractors for large eta/small W1..."

We agree with the reviewer. We now specifically refer to Fig.5 in the text here.

Line 474: see again note for line 191 – results showing BTSP have found the temporally asymmetry to be a defining characteristic. Need to explain why "temporally symmetric BTSP" is still BTSP.

We find nothing intellectually objectionable to taking the BTSP rule from CA1, choosing symmetric plasticity kernels, and studying its effect in recurrent networks. Our rule has all of the hallmarks of BTSP, with the sole exception of the asymmetry. Hence we call it temporally symmetric BTSP. As it turns out, this is precisely what one finds in CA3.

Line 481: as in, the time between environments, or the time between plateau events in a single environment?

The time between environments.

Line 564: repeat comment, but could all this same analysis be applied to a recurrent network encoding memories via other plasticity rules?

Well, if there were another plasticity rule which led to Eqs.13-15, then yes, the analysis would be the same. However, Eq.13-14 were derived from the BTSP rule and fit to the model from Milstein. Eq.15 is the extension of this rule to a recurrent network (note that the general formulation does not specify the shape

of the plasticity functions). Therefore our equations are directly linked to BTSP and not another plasticity rule. In fact, we cannot think of any other plasticity rule which would lead to Eqs.13-15.

Formatting and Presentation

In all figures: increase font size and line width where possible to improve readability.

Thank you. We have made changes wherever possible.

Fig 1: make use of colors consistent, indicate what colors mean in each panel. Also, panel E is a key part of the paper, but it is hard to understand. This didn't make sense to me prior to the methods. I think it is possible to convey using a more descriptive figure.

Ok with colors. We have added some more descriptive text to Fig.1E to improve clarity.

Fig 4: panel a) write out initial condition rather than "I.C."

Done.

Fig4: panel c and d) describe the vertical red and black dotted lines (panel D), and both vertical red horizontal black dotted lines in panel C. Figure caption for panel C refers to "dotted line" but unclear which one it is referring to.

Fixed.

Panel d) Since eta goes to 250, hard to distinguish the dots from each other - this is much more legible in figure 5b. Maybe only plot every 10 eta? star is confusing since it is at the same height and is of comparable size/same color as the elevated dot.

This panel is meant to show the projection of the bump solution onto all of the different manifolds (environments). If we were to leave any dots out, it would be unclear what the true projection was.

Panel e) clarifying that this is environment n-30 would help readability (both in panel and in figure caption), even though it is implied by the star in D.

Done.

Panel e) describe what the dots mean

Done

Panel f) show instances of the network in each of the three regimes (in the style of panel a). Also, if all three regimes exist for w0¿=0, why set it to be negative? Doesn't this add an extra assumption of inhibition which is unnecessary?

Panel A illustrated the fact that one could obtain bumps with different orderings, corresponding to different environments, in the same network. The three regimes of panel f are different. To the left, the activity is simply flat (no bump), in the bistable regime it can be flat or spatially modulated, and to the right only bump solutions exist. We do not think that plotting this activity will add anything here. Regarding inhibition, it is always present, even for $w_0 > 0$. The parameter w_0 indicates the net mean input weight. It is positive when excitatory inputs dominate, negative when inhibitory inputs dominate, and zero when they are balanced. In the absence of any inhibition, w_0 would be large and positive. In this regime, no bump solutions exist. We do not discuss this regime, because it is not of biological interest.

Fig 5: Unclear what "measured with different orderings" means. Do you mean measured in different environments (which have their unique ordering)? If so, pick one naming convention ("environment" is probably the most intuitive) and stick to it. If not, more description is needed to distinguish "measured with different orderings" and "measured in different environments"

The problem is that "measured in different environments" is not really correct. When we model the plasticity, it can be assumed that the animal is in a given environment. However, when we consider the intrinsic dynamics of the network, this is not necessarily the case. Therefore, it is more correct to say "measured with different orderings, corresponding to different environments". We have now changed the caption to make this clearer.

Fig 6: please show statistical significance, unclear to what degree the network actually outperforms the ring model approximation. Define what "optimal" means in "scales optimally" and compare panel B to classical memory networks.

The reviewer makes a good point regarding the performance of the network compared to the ring model. It was very hard to discern this in our original figure. In order to illustrate this more clearly we have now added an inset in which we show the fractional difference between the network and ring-model capacities, $(\eta_{max}^{network} - \eta_{max}^{ring})/\eta_{max}^{ring}$. The result is non-monotonic. Specifically, for non-sparse networks $s \sim 1$, the ring model approximation is not good, as seen in Fig.5, and the memory capacity of the network is actually less than that of the corresponding ring model. In the sparse coding limit, for small s the network outperforms the ring model. Why is this the case? We are not entirely certain, but our guess is the following. We know from our analysis that the quenched variability in the connectivity arising from the interference between stored memories actually enhances the memory capacity. It does this through contributions to the first spatial Fourier mode, which at some points along the ring aid in driving a bifurcation. This is similar to how temporal noise can drive oscillatory activity even below a Hopf bifurcation. This effect holds for both the network model, and the ring model, in which we explicitly include the quenched variability. However, there is an additional source of variability in the network model which is not included in the ring model. Namely, quenched variability

in the firing rates. In the network model, at each spatial position there are sM neurons, each of which is statistically equivalent, but which nonetheless have distinct dynamics and, in the steady state, distinct firing rates. The ring model simply assumes a mean rate at each position and hence cannot take this variability into account. We believe it is this additional variability which improves the capacity. It is not a small effect. For values of s below 0.2 there is between a 20 to 40% improvement in capacity. However, it seems that in the limit $s \to 0$ the network capacity once again approaches that of the ring model, leading to an optimal value of coding sparseness. We cannot be entirely sure of this, although we have added an additional data point now $s = 0.025$ (see also the main panels in Fig 6). Simulations for yet smaller s are currently prohibitive for us, but looking at this more in detail could be future work.

Regarding the scaling as $s \to 0$, the quadratic scaling is optimal from an information-theoretic standpoint. This is a classical result, and is shared by several classes of synaptic models in the sparse-coding limit, including: unbounded synapses (such as in a Hopfield net) and binary synapses (0s and 1s). Again, see the comprehensive review by Stefano Fusi (our reference [21]) for a detailed explanation (Table 1 in that paper).

Reviewer 2

The assumption that each cell induces plateau potentials may oversimplify the sparsity and randomness of PPs. Please provide justifications, references or some discussion for this assumption.

The reviewer is correct. In the end we focus on the sparse coding case $s \ll 1$ for which only a small fraction of the total network are place cells in any given environment. In that case the interpretation would be that plateau potentials (PP) have occurred in only a small fraction of cells. Namely, the likelihood of a PP would determine the coding sparseness. In the unrealistic limit of $s = 1$ this would require a PP in every cell, and this is the first case we consider. We have now written "In the more realistic sparse-coding case studied in the next section, PPs will only occur in a small fraction of cells, which will determine the sparseness."

Please provide more details on how BTSP constructs the connection weights and how it operates in firing rate models when simulating the network of firing rate neurons.

We model plasticity by simulating the 1D map directly. This is why we felt it was important to show that the map can accurately reproduce results from the biophysical model. When simulating the map, we do not explicitly consider any firing rate dynamics. The firing rate is, in part, implicit, because in the sparse coding case only a fraction of synapses are updated: those that connect active cell pairs. Once the synaptic weight matrix has reached a steady state, we being storing the different orderings of cells (and subsets of cell activations) for different environments. We do this until a sufficiently large number of environ-

ments have been stored (beyond the max capacity). The time to reach steady state, and the number of environments needed to be stored beyond steady state, depends on parameters, and particularly strongly on the sparseness s.

Once this is all done, then we use the resulting matrix for the firing rate simulations. Therefore, the plasticity process, and the firing rate simulations are completely separate processes. For the network simulations, we use the full weight matrix, with the noted normalization: pre-multiply by max weight and include the effect of inhibition. Then we simply carry out simulations and see what we get. In principle we need to look at the activity using every single stored ordering in order to know if the solution is correlated with one memory, several, or none. For the ring model simulations, we use the analytically calculated values for the recurrent connectivity for each ordering separately. We also include the normalization factor used in the network in order to compare the two quantitatively.

We have now added two sentences to the opening paragraph of the section *Neuronal networks endowed with BTSP...* to clarify the separation between plasticity and firing rate dynamics. They are: “In doing this, we have implicitly assumed that a subset of cells is active in any given environment, but we have not explicitly modelled the firing rate dynamics. Rather, we have directly solved the 1D map.” and “Specifically, we consider now the firing rate dynamics in a network in which plasticity has already occurred, and no longer allow for additional plasticity.”

Although the simplified rule aligns with the biophysical model, it is worth noting that the parameters used for simulations fall outside the range of experimental fitting (Milstein 2021 [10]). In fact, the heapmap of the change in weights shown in Fig.1G differs from that of (see Fig.3I of Milstein 2021). Considering that the biophysical model allows for nine free parameters, it would be beneficial to demonstrate that the model accurately fits the experimental curve suggested by previous studies.

This is a good point. We chose the parameters we did because they were the ones used in the original version of the Milstein et al preprint. Those authors then changed the parameters for the published version. In the published version, the parameters are fit separately to each cell recording. This includes the non-constant velocity of the animal for each run. Reproducing this data would be a major undertaking for us, and we do not feel that it is key to our contribution. However, it is true that if the biophysical model had completely different behavior for those parameter values, which cannot be captured by the 1D map, that would be a serious problem. We therefore now include heatmap comparisons, in Supplementary Fig.1, for parameter values from the ranges listed in Milstein 2021. Note that only the parameter *ranges* are given in Milstein et al, not the actual parameters sets used for each cell. So we cannot be sure how close to the parameters for any specific given cell we are. In any case, the heatmaps are very similar, although there are some quantitative discrepancies.

To play it safe, we have now removed the sentence from the abstract stating

that the map provides a quantitative fit to experimental data. We believe it can, but we do not have that exact data and have not made that fit! In this vein, (old) lines 55-56, we now state that we can *qualitatively* reproduce findings...

Since BTSP is primarily discovered in the connection between CA3 and CA1, it would be valuable if the authors could discuss the applicability of the proposed rule to CA3 as well.

Recent work from Jeff Magee's lab has answered this question for us. BTSP is present in CA3, it specifically shapes the recurrent connections between place cells, and is symmetric as we have assumed. (Li et al, biorxiv 2023.05.30.542781)
